# Generation of functional dopaminergic neurons from human spermatogonial stem cells to rescue parkinsonian phenotypes

**DOI:** 10.1186/s13287-019-1294-x

**Published:** 2019-06-27

**Authors:** Hao Yang, Dingjun Hao, Cheng Liu, Dageng Huang, Bo Chen, Hong Fan, Cuicui Liu, Lingling Zhang, Qian Zhang, Jing An, Jingjing Zhao

**Affiliations:** 1 Translational Medicine Center, Hong Hui Hospital, Xi’an Jiaotong University, Xi’an, 710054 China; 2 Department of Foot and Ankle Surge, Hong Hui Hospital, Xi’an Jiaotong University, Xi’an, 710054 China; 3 Department of Spine Surgery, Hong Hui Hospital, Xi’an Jiaotong University, Xi’an, 710054 China

**Keywords:** Human spermatogonial stem cells, Direct conversion, Dopaminergic neurons, Neurophysiology activity, cell transplantation, Parkinson’s disease

## Abstract

**Background:**

Recent progress in the induced generation of dopaminergic (DA) neurons from different types of stem cells or reprogrammed somatic cells holds tremendous potential for the treatment of Parkinson’s disease (PD). However, the lack of a reliable source for cell replacement therapy remains a major limitation in the treatment of human neurological disorders. Additionally, the current protocols for in vitro differentiation or cell reprogramming to generate human DA neurons are laborious, time-consuming, and expensive, and efficient conversion of human spermatogonial stem cells (hSSCs) to functional DA neurons has not yet been achieved.

**Methods:**

Primary hSSCs from testicular tissues of patients were exposed to an improved induction system, which consisted mainly of olfactory ensheathing cell conditioned culture medium (OECCM) and a set of defined cell-extrinsic factors and small molecules. Morphological changes were assessed, along with the expression of various DA neuron phenotypic markers (e.g., Tuj-1, TH, Nurr1, DAT) and several critical pro-DA neurogenesis effectors (e.g., EN-1, Pitx3, Foxa2, Lmx1a, Lmx1b, and OTX2). In addition, transcriptome analysis was used to further evaluate the genetic similarity between the artificially differentiated DA neurons and genuine ones. Concomitantly, the functional properties of converted DA neurons including synapse formation, dopamine release, electrophysiological activity, and neuron-specific Ca^2+^ signaling images were determined. Finally, hSSCs in the early stage of induction were evaluated for survival, differentiation, migration, tumorigenicity in the mouse striatum, and improvement of functional deficits in MPTP-induced PD animals.

**Results:**

The hSSC-derived neurons not only acquired neuronal morphological features but also expressed various phenotypic genes and protein characteristic of DA neurons and several effectors critical for pro-DA neurogenesis. Strikingly, as the period of induction was prolonged, expression of the critical molecules for DA neuron epigenetic status gradually increased while hSSC-specific markers sharply decreased. After 3 weeks of induction, the transdifferentiation efficiency reached 21%. In addition, hierarchical clustering analysis showed that the differentiated DA neurons closely resembled genuine ones. Furthermore, the hSSC-derived neurons gained sophisticated functional properties of wild-type DA neurons, and pro-induced hSSCs efficiently survived, migrated, and differentiated into DA neurons without tumorigenesis after transplantation into mouse striatum, leading to improvement of functional deficits in PD animals.

**Conclusions:**

The results showed that, using the present improved straightforward approach, hSSCs could acquire DA neuron morphological features and functional properties and rescue parkinsonian phenotypes. Our strategy for the conversion of hSSCs into DA neurons is very efficient and thus may provide an alternative approach suitable for clinical cell therapy to treat neurodegenerative diseases including PD.

## Background

Parkinson’s disease (PD) is the second most prevalent neurodegenerative disorders characterized by marked depletion of dopamine caused by the loss or degeneration of DA neurons in the substantia nigra (SN) of the midbrain [1–3]. Thus far, there is still no curative treatment for PD although existing treatments such as deep brain stimulation and pharmacotherapies (including levodopa monoamine oxidase B) can alleviate some of the symptoms; they tend to lose efficacy over time and thus do not prevent irreversible disabilities indefinitely [4–7]. Based on the molecular pathological properties of PD, cell replenishment therapy, especially for DA neurons, is considered the most attractive approach to treating PD patients. The advent of stem cells and cellular reprogramming has brought the gospel for PD patients, opening the way for potential applications of cell-based therapy. Among various types of cells, neural stem cells (NSCs) have long been demonstrated to be a promising candidate for treating PD. Nevertheless, the clinical application of NSCs is still challenging due to limited cell sourcing and ethical concerns. Therefore, other types of stem cells that are readily available appear to hold enormous promise for cell replacement therapy in regenerative diseases.

In various studies, reprogrammed somatic cells or stem cells including induced pluripotent stem cells (iPSCs), embryonic stem cells (ESCs), and mesenchymal stem cells (MSCs), e.g., from individual sources, have exhibited the unique characteristics of self-renewal capacity and multipotential differentiation. Nevertheless, each type has pros and cons as an available source for regenerative cell-based therapies. In particular, there are several serious concerns including ethical issues, tumor formation, and genetic instability, thus undermining their therapeutic application [8–10]. These are mainly attributable to the generation of iPSCs with several exogenous genetic manipulations, destroying the embryo in harvesting ESCs and generating teratoma arising from intermediate pluripotent cell state after transplantation [10, 11]. Additionally, current protocols for differentiating these types of stem cells into lineage-specific neurons such as DA neurons usually have poor conversion efficiency and are highly involved in terms of time consumption, technical complexity, and induction materials [12–14]. Therefore, it is imperative to seek an alternative stem cell source that can generate DA neurons while being free of evident disadvantages such as ethical issues and tumor formation. Meanwhile, it is of great importance to establish a highly efficient and cost-effective differentiation strategy to generate functional DA neurons from stem cells.

Spermatogonial stem cells (SSCs) are a subpopulation of type A spermatogonia [15, 16]. SSCs were previously regarded as unipotent stem cells, differentiating into sperm only. With further studies on SSCs, however, the previously prevailing orthodoxy has been challenged. To date, a growing number of studies have demonstrated that SSCs from both mouse and human testes can dedifferentiate into ES-like cells that are capable of giving rise to various cell lineages of all three germ layers [17–23], implying that SSCs may be an alternative cell source for regenerative medicine. In addition, SSCs have several merits over other types of stem cells, including lack of ethical issues, lower tumorigenesis, and no immunogenicity [24]. More notably, SSCs can transdifferentiate into uterine, prostatic, and skin epithelium cells in vivo after transplantation into these tissues, mainly due to the strong sensitivity of SSC to living microenvironments [21]. Thus, SSCs could serve as another potential source for replacing damaged or degenerated cells in various neurodegenerative diseases. However, direct conversion of hSSCs to functional DA neurons without forced expression of transcription factors or reprogramming to a pluripotent state has not been achieved in vitro.

Here, we describe a straightforward two-step induction strategy for differentiating hSSCs into DA neurons based on unique characteristics of SSCs. Using this protocol, we achieved the direct conversion of hSSCs into DA neurons that recapitulated the morphological, key biochemical, and functional features of primary midbrain DA neurons. Notably, the acquisition of hSSC-derived DA neurons using OECCM and a set of defined cell-extrinsic factors as well as several small molecules could be much safer than other options. On the other hand, the newly established differentiation protocol obviates a complicated manipulation procedure and reduces the overall cost of the differentiation process. The present groundbreaking study may open the possibility of generating human DA neurons from hSSCs for PD therapy.

## Materials and methods

### Preparation of primary hSSCs

Testicular tissues from obstructive azoospermic (OA) patients at the age of 25–45 years who had normal spermatogenesis and needed microdissection for testicular sperm extraction were fully washed in DMEM/F12 (Gibco) with antibiotics (Gibco). Importantly, all experiments were performed in accordance with the relevant guidelines and regulations of the Institutional Ethical Review Committee of Hong Hui Hospital affiliated to Xi’an Jiaotong University. After the seminiferous tubules were minced into small pieces with an iris scalpel, primary hSSCs were isolated using a two-step enzymatic digestion and subsequently purified as described in our previous studies [25]. For cell expansion, these purified hSSC suspensions were seeded into poly-lysine-coated 60-mm diameter tissue culture dishes in DMEM/F12 supplemented with 10% fetal bovine serum (FBS) (Gibco),100 ng/ml glial cell-derived neurotrophic factor (GDNF, R&D), 10 ng/ml basic fibroblast growth factor (bFGF, Sigma), 20 ng/ml epidermal growth factor (EGF, Sigma), and 10 ng/ml leukemia inhibitory factor (LIF, Sigma) and finally maintained at 34 °C with 5% CO_2_.

### Preparation of primary OEC conditioned medium

Since OECs exert an important role in neural cell survival, development, and differentiation and because the fate of SSCs is readily influenced by their microenvironments or niche, OECs were prepared to induce differentiation of hSSCs into special types of neurons. The OECs were cultured and purified according to the methods described previously [26]. For the harvest of OEC conditioned culture medium (OECCM), the purified OECs were subcultured and maintained in DMEM/F12 containing 1% G5 supplement in a humidified 5% CO_2_ incubator. When the OEC confluency reached 80%, half of the total cell supernatant was collected once every day, concomitantly centrifuged and filtered with a 0.45-μm pore membrane to remove cell debris. At 7 days after collection, these supernatants were fully mixed and stored at − 80 °C for the subsequent experiments.

### Differentiation of hSSCs

The protocol for transdifferentiating hSSCs into functional DA neurons included two steps. First, hSSCs were seeded at a density of 1 × 10^4^ cells/cm^2^ on poly-lysine-coated plastic coverslips or 6-well plates with OECCM containing 15 ng/ml GDNF (R&D Systems), 5 μM RA, 250 ng/ml SHH (R&D System), 1 ng/ml TGFβ3 (R&D System), 100 ng/ml FGF8α (R&D Systems), 1 mM VPA (Sigma), 10 μM SB (Sigma), and 1 μM forskolin (Sigma). The induction medium was changed every 2 days. After 4 days of induction, this medium was supplemented with 250 ng/ml SHH (R&D System), 1 ng/ml TGFβ3 (R&D System), 100 ng/ml FGF8α (R&D Systems) and maintained for another 3–4 weeks. Notably, at least 5 duplicate wells or coverslips were set up in each experiment. Thereafter, the cultures were observed and imaged under an inverted contrast microscope.

### Quantitative real-time PCR

Total RNA was extracted from primary hSSCs, fully transdifferentiated cells, brain tissue, hNSC cell line, and genuine cells using TRIzol Reagent (Invitrogen, Carlsbad, CA, USA). Reverse transcription (RT) of 2 μg of each total RNA was performed using the Primed Script™ Master Mix Kit (Takara) according to the manufacturer’s protocol. The selected target genes were as follows: GPR125, UCHL1, GFRA1, PLZF, GFRA1, Tuj-1, TH, Curr1, DAT, GFAP, CNP, Nestin, Pax6, CD133, EN-1, Pitx3, Foxa2, Lmx1a, Lmx1b, OTX2, TRA1-81, SSEA-1, PcNA, C-Myc, and GAPDH. The sequences designed for the gene primers are listed in Table 1. mRNA levels were quantified by SYBR green-based quantitative real-time PCR (Takara) using an ABi Prism 7900 HT (Applied Biosystems). The results were confirmed in at least 3 separate analyses.

**Table 1:** Primer sequences used for qPCR

Gene	Forward primer	Reverse primer	Product size (bp)	Tm (°C)
Tuj-1	GGCCAAGGGTCACTACACG	GCAGTCGCAGTTTTCACACTC	85	62
TH	GGGCTGTGTAAGCAGAACG	AAGGCCCGAATCTCAGGCT	107	61
DAT	TTTCTCCTCTCCGTCATTGGC	TGAAGCCCACACCTTTCAGTAT	226	62
Nurr1	ACCACTCTTCGGGAGAATACA	GGCATTTGGTACAAGCAAGGT	175	61
GFAP	CTGCGGCTCGATCAACTCA	TCCAGCGACTCAATCTTCCTC	209	61
CNP	AACAAGGCTTCTCCCGAAAA	GTCTTCACTCTAGCAGCGT	149	62
Nestin	CTGCTACCCTTGAGACACCTG	GGGCTCTGATCTCTGCATCTAC	141	62
CD133	AGTCGGAAACTGGCAGATAGC	GGTAGTGTTGTACTGGGCCAAT	99	62
Pax6	TGGGCAGGTATTACGAGACTG	ACTCCCGCTTATACTGGGCTA	111	61
GPR125	GCGTCATTACGGTCTTTGGAA	ACGGCAATTCAAGCGGAGG	199	60
Plzf	GAGATCCTCTTCCACCGCAAT	CCGCATACAGCAGGTCATC	139	61
DAZL	GCCCACAACCACGATGAATC	CGAGGTACAACATAGCTCCTTT	163	61.5
GFRA1	CCAAAGGGAACAACTGCCTG	CGGTTGCAGACATCGTTGGA	121	61.5
UCHL1	CCTGTGGCACAATCGGACTTA	CATCTACCCGACATTGGCCTT	201	62
EN-1	CGCAGCAGCCTCTCGTATG	CCTGGAACTCCGCCTTGAG	174	62.2
Pitx3	CAGAGGACGGTTCGCTGAAAA	AGCTGCCTTTGCATAGCTCG	240	62.7
Foxa2	GGAGCAGCTACTATGCAGAGC	CGTGTTCATGCCGTTCATCC	83	61.9
Lmax1a	ACGTCCGAGAACCATCTTGAC	CACCACCGTTTGTCTGAGC	248	61.5
Lmax1b	CGGACTGCGCCAAGATGTT	TTGACTCGCATCAGGAAGCG	160	62.5
OTX2	TATCTAAAGCAACCGCCTTACG	GCCCTAGTAAATGTCGTCCTC	134	60.2

### Immunofluorescence

Immunofluorescence staining was performed to determine the identity of cultured hSSCs and the differentiated hSSCs by the induction system pursuant to the procedure as described previously [27]. Briefly, cells on coverslips or plates were fixed in 4% paraformaldehyde for 20 min, permeabilized with or without 0.01% Triton X-100 (Sigma) for 10 min, and blocked in 4% normal donkey or goat serum at room temperature. Concomitantly, cells were incubated with primary antibodies at a dilution of 1:200–1000, respectively, overnight at 4 °C. The primary antibodies used in this study were as follows: UCHL1 (AbDSerotec), GFRA1, EN-1, OTX2 (Santa Cruz), GPR125, Tuj-1, PLZF, FoxA2 Lmx1a, Lmx1b (Abcam), DAT (Millipore), Curr1 (Millipore), PitX3 (GeneTex), and SYN (Chemicon). After three washes in PBS, the cells were incubated with corresponding Alexa Fluor® 488, Alexa Fluor® 594, or FITC-conjugated-IgG secondary antibodies at a 1:200–1:500 dilution for 1 h at RT. DAPI (4″-6-diamidino-2-phenylindole) was used to stain cell nuclei, and the cells were observed under a fluorescence microscope (Olympus BX-53, Japan).

### Western blots

Western blot analyses were performed as previously described [28]. Antibodies were used to stain specific proteins: Tuj-1, TH, Curr1, DAT, Synapsin, VASA, GPR125, PLZF, and β-actin. β-actin was used as an internal loading control. Blot densitometric analysis was repeated in triplicate, and the integrated density values (IDV) of the γ-tubulin standard were calculated.

### Transcriptome analysis

Total RNA was extracted from isolated hSSCs, differentiated cells, and wild TH^+^ cells using an RNeasy micro kit, then quantified and hybridized to each microarray chip. RNA (10 ng) was reverse transcribed into first-strand cDNA using a T7-Oligo (dT) Primer (Two-Cycle Target Labeling and Control Reagent package, Affymetrix, CA, USA). After second-strand cDNA synthesis, the double-stranded cDNA was purified and served as a template in the first cycle of the in vitro transcription reaction. The following procedures were undertaken as described previously [29–31]. The labeled cRNA was cleaned up, fragmented, and hybridized to the human genome. A total of six arrays (three each for hSSCs, TH cells, and differentiated cells) were used in the present study. The arrays were stained according to the manufacturer’s protocols and then scanned with a GeneChip scanner (Affymetrix, CA, USA). Initial analysis of the scanned images was performed by using GeneChip Operating Software (GCOS, Affymetrix, CA, USA). For absolute analysis, each chip was normalized to a target intensity of 500, and probe sets were assigned a signal intensity and detection call of present, marginal or absent.

### Cell count

To determine the transdifferentiation efficiency of hSSCs to DA neurons under the induction conditions mentioned in the previous section, all TH-positive cells and Tuj-1-positive cells, regardless of their morphology, were counted as described previously [28]. Notably, the total transdifferentiation efficiency of hSSC was assessed by the ratio of TH^+^ cells and DAPA labeling cells or Tuj-1^+^ cells. Cell count was performed by a person blinded to the experimental setting. From each coverslip 15 randomly chosen fields were counted. In all analyses, data represent the mean ± SEM of 3 independent experiments. Each performed experiment was comprised of 5 coverslips and the obtained result considered as the transdifferentiation efficiency index.

### Electrophysiology

After induction of hSSCs for 3 weeks, the differentiated cells were transferred to artificial cerebrospinal fluid (ACSF). The ACSF was made as previously described [24]. Tetrodotoxin (TTX, 0.4 μM), tetraethylammonium chloride (TEA, 0.5 μM), and bicuculline (BIC, 10 μM) (Sigma) were diluted in the bath solution and applied via gravity using an SF-77B perfusion fast-step system as described previously [32]. Whole-cell patch clamp experiments were performed at 20 °C to 22 °C under an inverted microscope (Zeiss, Jena, Germany). Cells with leak currents < 100 pA were used for further analysis. Whole-cell currents were low-pass filtered at 2.9 kHz, digitized at 10 kHz using an EPC-10 amplifier (HEKA, Lambrecht, Germany) and analyzed with Patch Master (HEKA).

### Calcium imaging

To determine whether hSSC-derived neuronal cells are functional, we assessed the calcium influx of individual cells as previously described [33, 34]. Briefly, after induction for 3 weeks using the special induction system, the differentiated cells were incubated with 2 μM of the calcium ion-sensitive fluorescent indicator Fluo-2 AM (Sigma) in Neurobasal medium containing 1% B27 for 30 min at 37 °C. After the slices were fully washed twice with extracellular medium (EM, 140 mM NaCl, 2 mM CaCl_2_, 5 mM KCl, 10 mM HEPES, and 10 mM glucose, adjusted to pH 7.2–7.4) twice, calcium imaging was conducted at 37 °C in EM with confocal Olympus microscopy with perfusion system. To specifically determine the Ca^2+^ influx, a Ca^2+^ channel activator (10 μM BayK, Stemgent) or blocker (5 μM nifedipine, Abcam) was added to cultures to observe the change in Ca^2+^ influx images. The entire procedure was executed according to our previous report [33].

### Dopamine release

Dopamine release was quantified using ELISA kits (Dopamine Research EIA, Labor Diagnostika Nord GmbH & Co. KG, German) analyses after 1, 2, and 3 weeks of differentiation in vitro. Briefly, the culture supernatants of induced hSSCs at different time points were collected and concentrated. In parallel, differentiated LUHMES, a type of human mesencephalic dopaminergic neuron line, was used as positive control of native DA expression. Their culture supernatants were treated as that of induced hSSCs. Subsequently, an enzyme immunoassay was performed to measure dopamine levels according to the manufacturer’s instructions. Each sample and standard was performed in duplicate. Absorbance was read using a microplate reader set to 450 nm and a reference wavelength set between 630 nm. Data were from three independent experiments.

### Animal model and surgery

To further investigate the neural conversion of hSCCs and their functions in vivo, we transplanted pro-induced hSSCs into the striatum of an animal model of PD. The animal model of PD was developed according to a previous method described [35]. Subsequently, 20 symptomatic male Balb/c mice (25–30 g) were used as graft recipients and were housed on a 12-h light/dark cycle with ad libitum access to food and water. All surgical procedures were conducted under general anesthesia using sodium pentobarbital solution injected intraperitoneally (i.p.) as described in detail [36]. For transplantation, a total of 3 μl of cell suspension was transplanted into the striatum of each animal at a concentration of 100,000 cells/μl on a stereotaxic apparatus. Thereafter, animals were injected i.p.15 μg/g ciclosporin A daily for immunosuppression. Additionally, antibiotic treatment was maintained in vivo by injection daily (1 mg/ml P/S). Importantly, all procedures were conducted in accordance with guidelines set by the Ethical Committee for the Use of Laboratory Animals at Xi’an Jiao Tong University.

### Behavioral assessments

For evaluation of the efficacy of hSSC-derived neurons of potential therapeutics, we conducted behavioral measures of the PD mouse model after cell transplantation. The behavioral tests, including the cylinder test, adhesive removal test, gait analysis, and ledged beam test, are relatively safe, easily performed and have extremely high sensitivity to subtle alterations in dopamine functions. These behavioral measures were performed as described previously [37, 38]. Notably, only mice that displayed stable or prominent symptoms were used for behavioral tests, and assays were performed at a minimum of four time-point posttransplantation, starting at 3 weeks. Eight mice with confirmed behavioral alterations were selected for each of various groups (sham group, hSSC groups). In addition, a short period of training was required to make animals to habituate the testing environment or certain equipment prior to behavioral experiments.

#### Cylinder test

To evaluate locomotor asymmetry in mouse models of PD after hSSC transplantation, we placed the animals in an open-top clear plastic cylinder with a diameter of 30 cm and height of 25 cm, and their forelimb activity while rearing against the wall of the arena was recorded as described previously [38]. In the test experiment, forelimb used is defined by the placement of the whole palm on the wall of the arena. The activity of each mouse was recorded for 3 min every time and conducted for 5 times daily and totally for 3 days. Notably, the testing area must be quiet as loud noises and conversation can distract the mouse and potentially cause freezing behavior. The functional locomotor assessment of 8 mice with PD model was carried out just over 3 weeks after cell transplantation.

#### Adhesive removal test

An adhesive removal test was used to detect the response to sensory stimuli and motor performance and coordination. The test was carried out according to a previously described method [38]. One adhesive label was gently pressed on the snout of the mouse using a pair of small forceps; subsequently, the time when the mouse attempted to remove the label with its forepaws was recorded. Importantly, the test was conducted in a clean home cage for every mouse. All 8 mice received 3 trials.

#### Gait analysis

To assess motor function and coordination by gait analysis in the PD animal model after hSSC transplantation, we painted the forelimbs and hindlimbs of the tested mice and placed them on a wooden platform with width of 5 cm and length of 50 cm, covered with an ink-absorbent paper, toward a little dark shelter located at end of the platform. To characterize the animal locomotion, we measured and calculated the walking distance during the 2-min walking test using the footprints made on the paper when the mouse walked. Additionally, the time it took for the animal to complete the walking task is recorded. Finally, the velocity was evaluated from animal walking time and total distance. Gait-related parameters—such as stride pattern, individual walking speed, and distance duration were reported for each animal. Of note, the functional gait assessment of total of 8 mice was carried out after hSSC transplantation.

#### Ledged beam test

To assess the improvement of sensorimotor deficits in the PD animal model after hSSC transplantation, we placed the tested mice on a ledged beam with the widest start to narrowest end (gradually tapered from 6 to 1 cm) and observed their movements along the beam to the desired direction. The procedures were conducted according to the previously described methods. Notably on the test day, all mice (8 mice each time) underwent 5 pretrials on the grid-surfaced beam to familiarize themselves with the test environment.

### Tissue analysis

Three, four, and five months after transplantation, the animals were perfused with ice-cold PBS and 4% paraformaldehyde (PFA) in PBS (pH 7.4). The brains were collected and postfixed overnight at 4 °C. Postfixed brains were cryoprotected with 30% sucrose solution in PBS and cryosectioned into 15 μm and mounted on gelatinized glass slides. Immunohistochemistry was performed essentially as previously described. Briefly, all brain sections were blocked with 2% BSA in PBS and permeabilized with 0.2% Triton X-100. Sections were processed for three markers (TH 1:500; DAT 1:800; HN 1:1500) to determine the cellular phenotype of the grafted cells. After incubated with respective fluorescent secondary antibodies diluted in blocking solution for 2 h at RT, nuclei were counterstained with DAPI for 15 min. Thus, sections were mounted onto glass slides for microscopic analysis following extensive rinsing with PBS. Notably, the stereological count to total HNu-positive cells and double-labeled cells with TH was conducted using a modified version of the optical fractionator method as reported by Corti et al. [39]. To further investigate in detail whether the in vivo differentiation of pre-induced hSSCs is likely to undergo ESC process, their transdifferentiation transition was elevated by means of double-immunostaining for human nestin (1300) and CyclinD1 (1200) 1–3 weeks after injection. Preparation of tissue sections and subsequent immunostaining were done according to the aforementioned procedure. Repeated measure ANOVA was used for statistical analyses.

### Amphetamine-induced rotations test

All animals were tested with amphetamine (2.5 mg/kg, Sigma) at 2 weeks after MPTP injection. Notably, only animals showing PD typical symptom were chosen and available for test. At 1, 2, and 3 weeks posttransplantation, animals were given amphetamine and the total rotations were quantified using automated rotometer bowls as described previously [40]. The rotational behaviors were assessed for 90 min with a video camera and full 360a second metham. Values are expressed as means ± SEM. Both contralateral rotations and ipsilateral rotations are counted.

### Statistical analysis

All of the data are represented as the mean ± SEM from at least three independent experiments. Statistical analysis was performed using the statistical software SPSS 10.0. Cell count data and behavioral assessment were evaluated statistically using repeated measures of ANOVA (analysis of variance), and *p <* 0.05 was considered statistically significant.

## Results

### Derivation of induced dopaminergic neurons

**Fig. 1:**
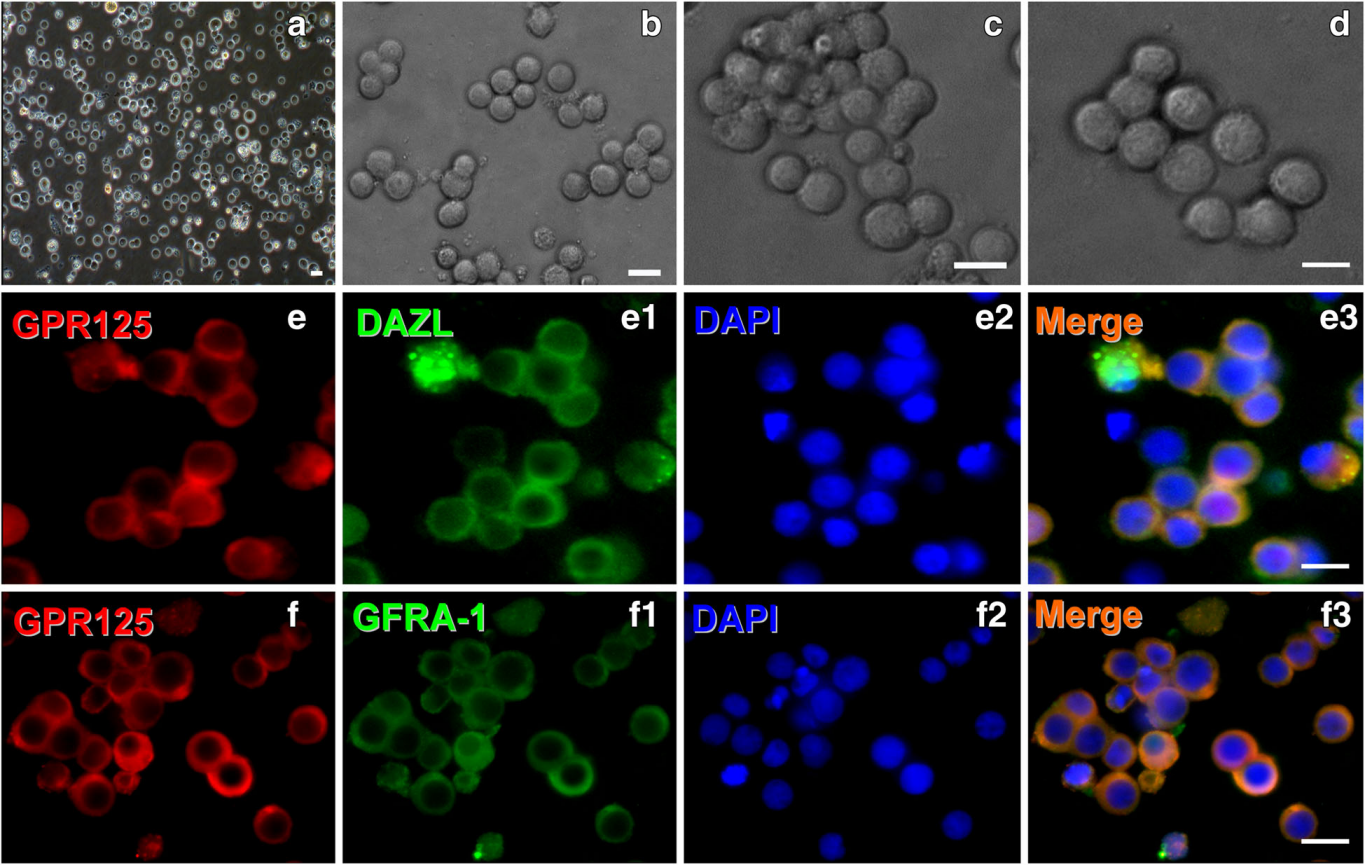
Morphological and biochemical features of primary hSSCs. Phase-contrast microscopy showed the morphology of the enzymatically dissociated cells (**a**) and purified hSSCs for 3, 5, and 7 days, respectively (**b**–**d**). **e**, **f** Immunofluorescence revealed the expression of GPR125, DAZL, and GFRα1 in primary hSSCs. Scale bars in **a**–**f** = 10 μm

Spermatogonial stem cells (SSCs) were prepared from human testicular tissues according to our previously reported method and further identified by morphological features and immunostaining with hSSC-specific markers. Phase-contrast microscopy showed the morphology of enzymatically dissociated (Fig. 1a) and purified hSSCs, respectively (Fig. 1b–d). Nearly all cells after purification stained positive for GPR125, DAZL, and GFRA-1, three specific markers for hSSCs, albeit more than third subcultures before identification (Fig. 1e, f). Under this culture condition, neither Sertoli cells nor neural cells were detectable, excluding the possibility of non-hSSC contamination in the cultures.

### Generation of dopaminergic neurons from hSSCs under defined conditions

**Fig. 2:**
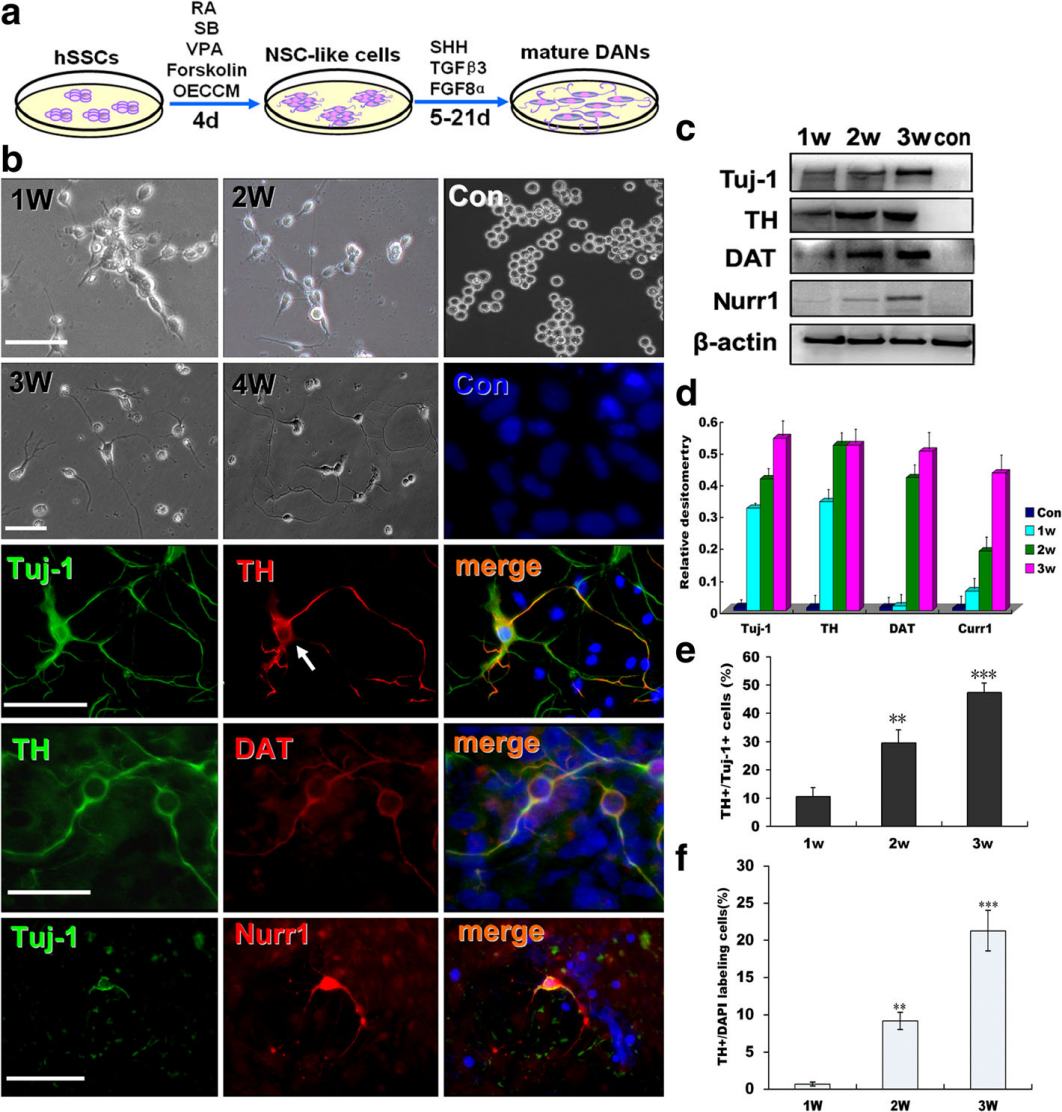
Direct conversion and characterization of DA neurons from hSSCs. **a** Schematic illustration of the experimental setup and strategy to convert hSSCs into DA neurons. **b** Bright-field hSSC (upper panels) images in induction medium for 1–4 weeks and immunofluorescence staining for DA neuron markers Tuj-1 TH, DAT and Nurr1 at week 3 (low panels). DAPI counterstaining (blue) was used to visualize nuclei. **c** Western blot analysis demonstrates a strong upregulation of the prototypic DA neuron markers Tuj-1, TH, DAT, and Nurr1 in hSSC-derived cells. β-actin was used as a loading control. **d** Quantitative analysis of Western blot results. **e** Percentages of Tuj-1^+^ and TH^+^ cells in hSSCs under this special induction condition for 1–3 weeks. **f** Total transdifferentiation efficiency of hSSCs after 1–3 weeks of induction. Data are reported as the means ± SEM of three independent experiments (**p < 0.01, ***p < 0.001)

We hypothesized that OECCM in combination with some small molecules known to drive cell phenotype changes in reprogramming and instruct DA neuron formation during development might facilitate the conversion of other cell types into DA neurons. To test this idea, in addition to OECCM, we first used four candidate molecules that participate in varied stages of cell phenotype transition, and then three factors (SHH, TGF8α, and TGFβ3) critical for cell specification were selected for further induction. To clarify the conversion of hSSCs to neurons, an experimental outline is shown in Fig. 2a. Next, we assessed morphology and biochemical phenotypes of the converted cells to confirm their identity after they had undergone special induction. As shown in Fig. 2b, after 1 to 4 weeks of induction, hSSCs underwent morphological changes from round to spindle-shaped or angular bodies with strong refractivity and enhanced stereo perception, gradually sprouted neurites and lost their own morphological profiles, clearly assuming a typical neural cell morphology. For normal hSSCs, no morphological changes were found. Encouragingly, a large number of cells expressed the pan-neuronal marker Tuj-1 and the DA neuron markers TH, DAT, and Nurr1 but not in the control at 3 weeks postinduction. In accordance with immunostaining, Western blots further revealed that the converted cells can express the abovementioned neuronal markers, and the expression of 4 molecules was time-dependent (Fig. 2c). Meanwhile, quantification also showed that there was a significant increase in their protein levels between the induction and noninduction groups (Fig. 2d). In addition, the conversion efficiency (the ratio of TH^+^ cells and DAPI labeling cells or Tuj-1^+^ cells) gradually increased within this induction duration (Fig. 2e and f), further suggesting that the differentiated cells acquired the characteristics of mature DA neurons in phenotypes under the appropriate induction conditions.

### Phenotypic characteristics and global gene expression of converted hSSCs

**Fig. 3:**
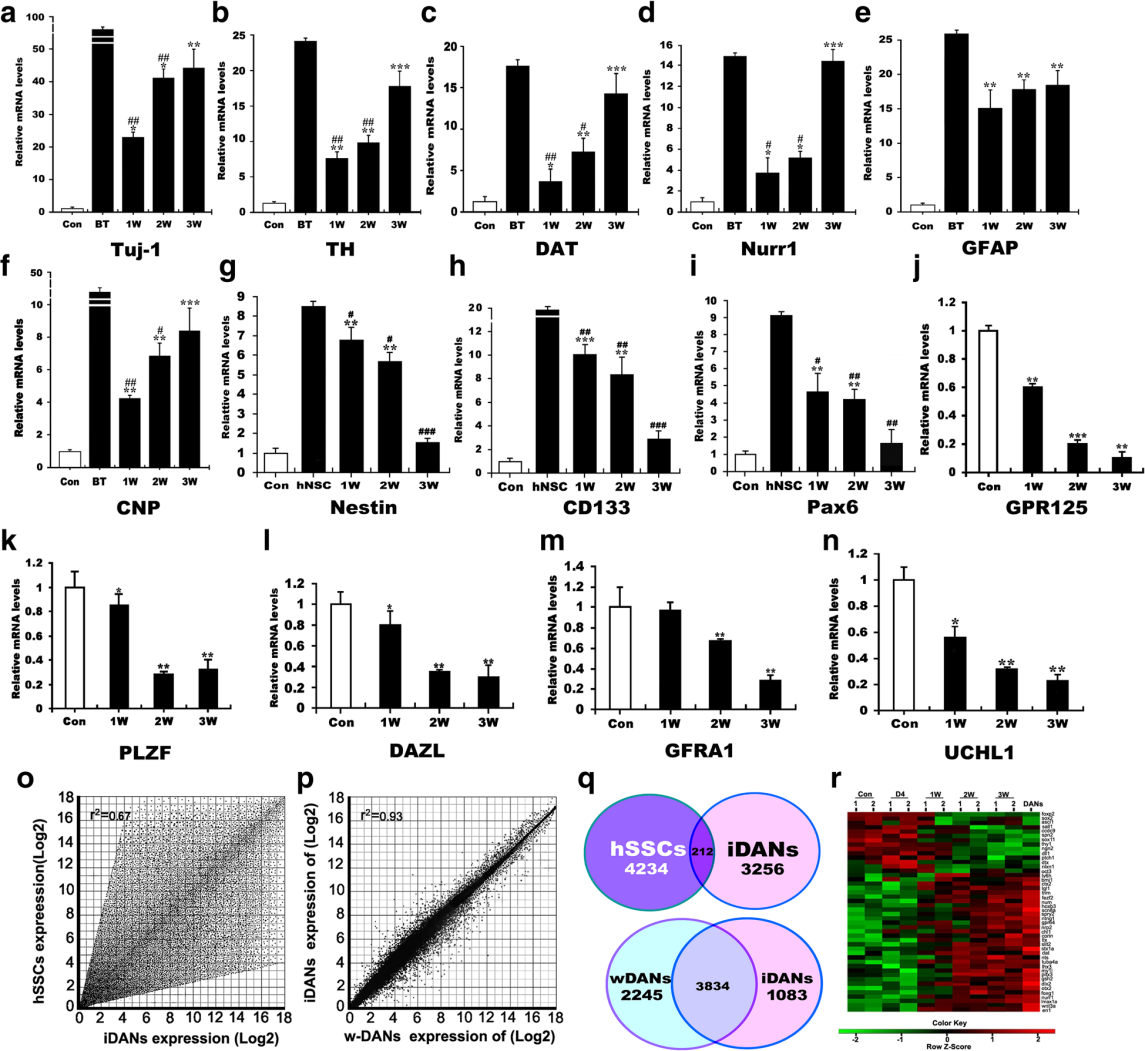
Phenotypic characteristics and global gene expression analyses of hSSC-derived DA neurons. qRT-PCR analyses showed the transcripts of neural markers Tuj-1 TH, DAT, Nurr1 GFAP, and CNP (**a**–**f**); neural stem cell markers nestin, Pax6, and CD133 (**g**–**i**); and hSSC markers PLZF, GPR125, DAZL, and GFRA1 (**j**–**n**) in hSSCs in induction medium for 1, 2, and 3 weeks. GAPDH was used as a loading control for total RNA. BT, brain tissue, was used as a positive control. “*” indicated statistically significant differences (*p < 0.05) compared to the control, while “^#^” denoted statistically significant differences (^#^*p* < 0.05) compared to the positive control. ** and ^*##*^ represent *p <* 0.01; *** and ^*###*^ represent *p <* 0.001. Three independent experiments are represented. **o**, **p** Homogeneity of gene expression visualized by scatter plot presentation. Shown are plots of the averaged intensities of each group as indicated. **q** Venn diagram of differentially expressed genes shared between hSSCs, hSSCs-derived DA neurons (iDANs) and w-DA neurons (w-DANs). **r** Hierarchical clustering analysis showed the global gene expression profiles of hSSCs undergoing this induction for different times

To address whether this special induction is sufficient for the transdifferentiation of hSSCs into DA neurons, we examined the various critical genes for the transition of hSSCs to DA neurons at transdifferentiation weeks 1, 2, and 3 by quantitative PCR analysis. As shown in Fig. 3a–i, after the indicative induction time points, the expression of neural cell markers (Tuj-1, TH, DAT, Nurr1, GFAP, and CNP) was progressively elevated within 3 weeks while the levels of NSC markers (nestin, CD133, and Pax6) were dramatically increased at 1 week and subsequently decreased. Compared to their corresponding controls, there were significant differences in the expression of these markers. Over 3 weeks of neural transdifferentiation of hSSCs in vitro, no remarkable difference was found between the differentiated cells and positive control (brain tissue), suggesting that these cells have acquired an identity. In contrast to the expression of the aforementioned neural markers, very lower expression of NSC markers (nestin, CD133, and Pax6) was also shown 3 weeks posttransplantation as compared to that of 1 and 2 weeks. There is a markedly statistical difference between the differentiated cells and positive control (hNSCs) (*p < 0.001*). To further substantiate our claims, we also measured the expression of hSSC markers (GPR125, PLZF, DAZL, GFRA1, and UCHL1) in the converted cells. Our results showed that the expression of these hSSC-specific markers sharply decreased for the duration of induction, and even disappeared, suggesting the validity and effectiveness of our induction method. Next, we performed an analysis of global gene expression and identified distinguishing genes between hSSCs and induced DA (iDA) neurons, as well as wild DA (w-DA) neurons and iDA neurons, respectively, and further compared global transcriptomic profiles of them. Of note, scatter plots and vena of gene expression analysis revealed that numerous genes were upregulated or downregulated in expression levels in the iDA neurons and that iDA neurons in the global gene expression pattern were highly distinguished from hSSCs but were similar to w-DA neurons. In addition, the expression of numerous genes in iDA neurons showed higher similarity to that in w-DA neurons (Fig. 3o–q). Notably, the 7490 genes showed an expression difference between iDA neurons and hSSCs, only 212 genes are conserved (Fig. 3q, upper panel); however, iDA neurons had established a program of w-DA neuron gene expression as they expressed many of same genes as w-DA neurons through transdifferentiation. Many of the 3834 known genes related to DA neuron development and differentiation, exhibited their important biological significance. At this time, 3328 genes showed an expression difference (Fig. 3q, lower panel). In addition, hierarchical clustering analysis showed progressively increasing similarities in the global gene expression profiles of the iDA neurons and w-DA neurons from 4 days to 3 weeks after induction, with especially strong similarity in gene expression properties at 3 weeks (Fig. 3r). In conclusion, these data indicate that the iDA neurons we produced had acquired characteristics of DA neurons.

### Activation of proneurogenic factors responsible for DA lineage specification

**Fig. 4:**
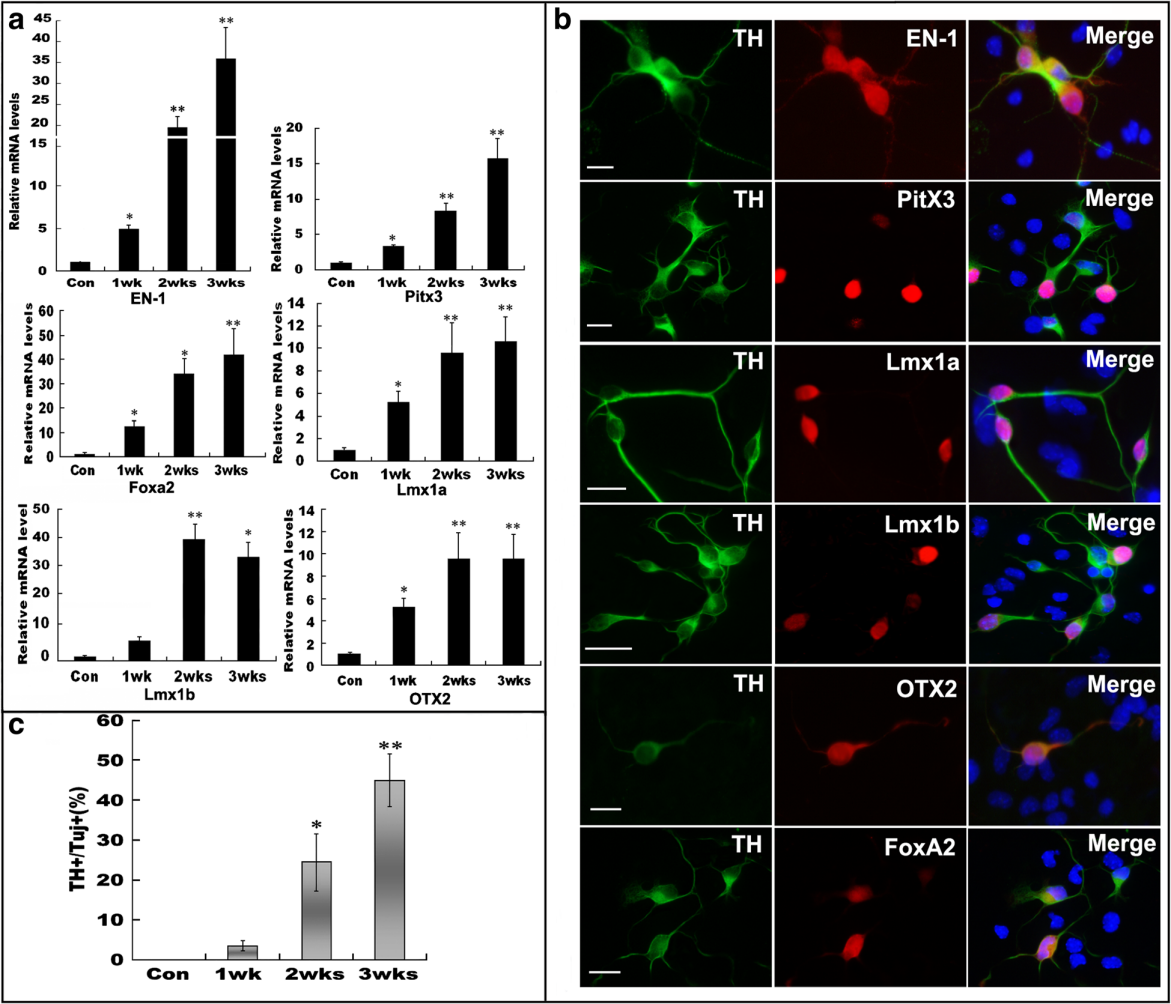
Activation of proneurogenic factors is necessary for DA lineage specification. **a** Quantitative RT-PCR analysis of genes essential for pro-neurogenesis DA lineage specification at the indicated induction time (1, 2, and 3 weeks). **b** Immunofluorescence revealed the expression of these indicated molecules in TH-positive cells induced by this special condition. **c** The yield of DA neurons with prolonged culture time. All data are reported as the means ± SEM. “*” and “**” represent *p <* 0.05 and *p <* 0.01, respectively, vs corresponding controls. Three independent experiments are represented. Scale bars = 10 μm

To characterize the transdifferentiation in great detail, we examined several crucial factors that initiate and drive the neuronal conversion of hSSCs and further DA lineage specification. We found that during the transdifferentiation of hSSCs to TH-expressing neurons, several proneurogenic factors (EN-1, Pitx3, Foxa2, Lmx1a, Lmx1b, and OTX2) were further upregulated significantly by combining OECCM induction with SHH, FGF8a, RA, forskolin, and GDNF(Fig. 4a). At longer induction times, especially week 3 of induction, the levels of these molecules in differentiating hSSCs were 10- and 40-fold higher than those in cells weeks 1 and 2, respectively. In contrast, these proneurogenic factors and DA lineage specification factors were not detectable in normal hSSCs at various time points. Consistent with the qRT-PCR results, immunostaining also showed that longer induction increased the expression of several DA lineage specification factors, particularly, EN-1, Pitx3, and Lmx1a (Fig. 4b). In addition, a higher proportion of TH^+^/Tuj-1^+^ DA neurons was yielded with longer induction (Fig. 4c). These results suggest that the special induction conditions truly initiate a neurogenic program and DA lineage specification.

### Formation of functional synapses and release of dopamine by hSSCs-derived TH-positive cells

**Fig. 5:**
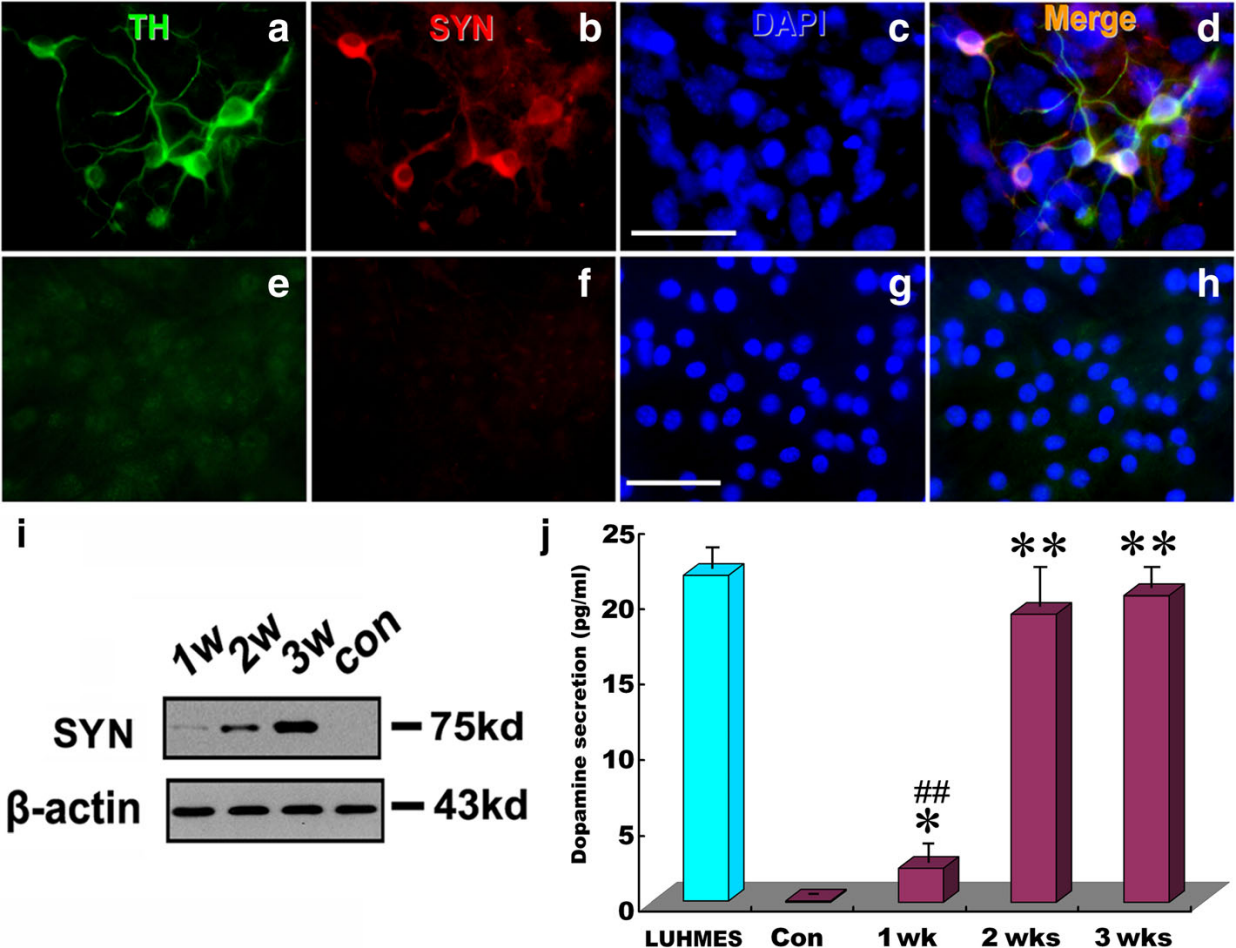
Functional assays of acquisition of mature phenotype. **a**-**d** Immunostaining of hSSC-derived neurons for TH (**a**) and synapsin (**b**) after 3 weeks of differentiation. Notably, DAPI staining (**c**) was used to visualize nuclei. **e**–**h** Immunostaining of hSSCs for TH and synapsin. **i** Western blot analysis of synapsin expression in hSSC-derived DA neurons after induction for 1, 2 and 3 weeks, respectively. β-actin served as a loading control. **j** Dopamine production of hSSCs in induction medium for 1, 2 and 3 weeks, respectively. Data from three independent experiments are reported as the means ± SEM. “^*##*^” and “**” represent *p <* 0.01 vs corresponding controls. Notably, “^*##*^” compared to LUHMES, a type of human mesencephalic dopaminergic neuron line, as a positive control of native DA expression. Scale bars = 50 μm

Our initial results indicated that hSSC-derived cells possess many of the biological phenotypic properties of DA neurons and undergo activation of several critical molecules responsible for dopaminergic neurogenesis. However, the defining functional characteristic of DA neurons is their ability to form synapses and release dopamine. To test whether the hSSC-derived cells could have the ability, we further determined the expression of synapsin, a hallmark of functional neurons, and the release of dopamine. Immunostaining indicated that the converted TH^+^ hSSCs expressed synapsin (Fig. 5a–d). In contrast, synapsin expression was not detectable in normal hSSCs (Fig. 5e–h). Consistent with immunostaining, Western blot analysis also revealed a remarkable expression of synapsin in converted hSSCs, and the expression levels of synapsin progressively increased with prolongation of induction time (Fig. 5i). Of note, a significantly higher release of dopamine was found in hSSC-derived neurons at 1, 2, and 3 weeks than in uninduced hSSCs, and the amount of dopamine secretion from hSSC-derived neurons was gradually increased with longer induction (Fig. 5j). It is noteworthy to mention that no significant difference was found in the amount of dopamine released from hSSCs induced between 2 and 3 weeks. Also, no significant difference in dopamine release between differentiated LUHMES and the induced hSSCs for 2 and 3 weeks was found. These results suggest that the hSSC-derived cells were mature and had functional attributes of DA neurons.

### Neurophysiological properties of DA neuron-like cells from hSSCs

**Fig. 6:**
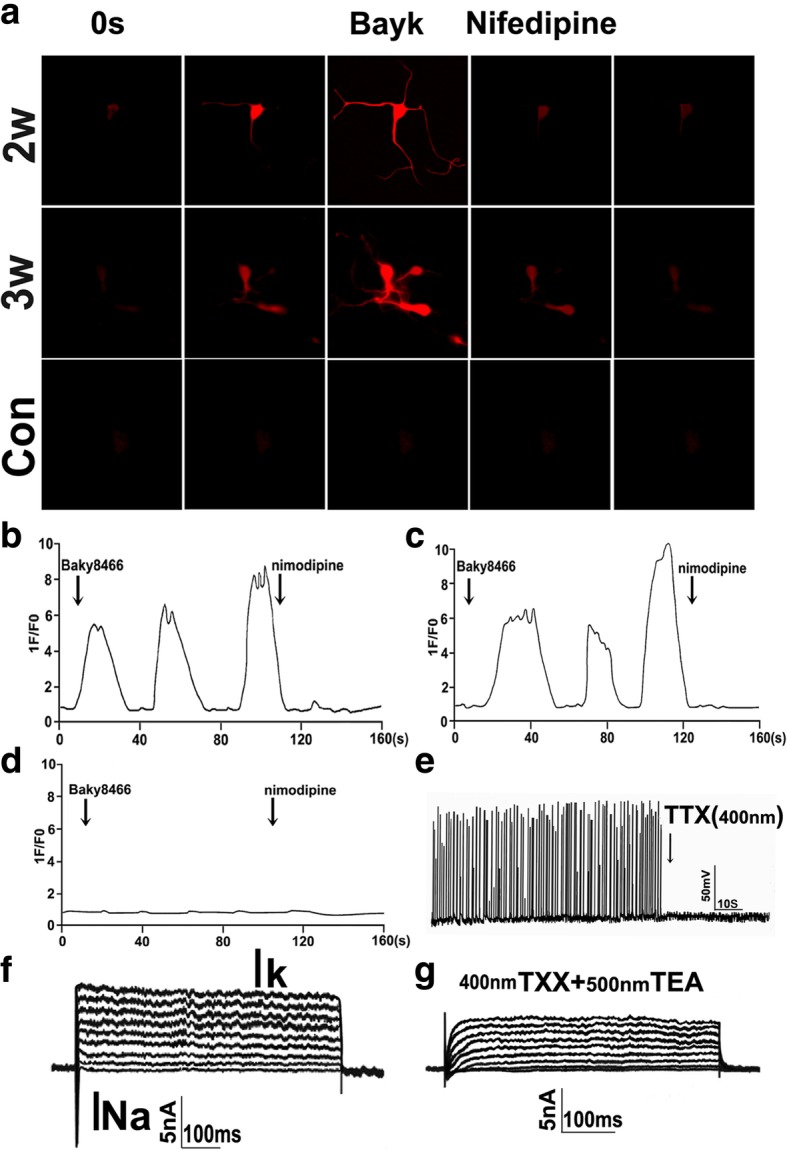
Physiological properties of neurons derived from hSSCs. **a** Functional characterization of the L-type calcium channel. The panels show typical calcium response imagines of hSSC-derived cells at 2–3 weeks postinduction.1F/F0 represents the ratio of fluorescence intensity of cells for 0 s and at the indicated time. BayK (10 mM) in the presence or absence of nifedipine (5 mM) was administered at the indicated time point. **b**–**d** Representative photographs of Ca^2+^ imaging of differentiated neurons or hSSCs in the presence or absence of nifedipine after treatment with BayK. Approximately 50 cells were tested in each experiment; the representative results are shown. Scale bar = 50 μm. **e** Current-clamp recordings of action potentials evoked in SSC-derived neurons after 3 weeks of induction. Action potentials were suppressed by TTX. **f** Voltage-clamp recording of hSSC-derived neurons. Depolarized sodium and potassium currents were evoked by the membrane potential to different levels. **g** Sodium and potassium currents were attenuated by sodium and potassium channel inhibitors TTX (400 nm) and TEA (500 nm), respectively

To further substantiate whether hSSC-derived cells truly have a functional activity after 3 weeks of differentiation, we assessed another most important indicator of functional neurons, i.e., neuron-specific calcium imaging. Our results revealed that after 2–3 weeks of differentiation, hSSC-derived cells can display an increased neuron-specific calcium influx as demonstrated by a drastic increase in fluorescence intensity, respectively, when treated with BayK, a calcium channel agonist. The fluorescence intensity rapidly decreased after the withdrawal of BayK and blocked by a specific calcium channel blocker, nifedipine. In contrast, there was not a remarkable increase in fluorescence intensity in hSSC cultures in the presence of BayK. As for hSSCs, the fluorescence in cells was hardly detectable (Fig. 6a). The quantitative analysis further indicates that there is an approximately 5- to 8-fold (2 weeks of induction) or 6- to 10-fold (3 weeks of induction) increase in fluorescence intensity, respectively, upon treatment with BayK. Notably, these converted cells displayed 2- to 4-fold stronger fluorescence intensity after the withdrawal of BayK and blocked by nifedipine (Fig. 6b, c). Fluorescence intensity reflecting Ca^2+^ flux in cells had no change regardless of the presence, withdrawal of BayK or addition of nifedipine in cultures (Fig. 6d).

To more definitively test whether hSSC-derived DA neurons display similar electrophysiological properties to genuine midbrain DA neurons, we performed patch-clamp recording in converted neurons from derived hSSCs at 3 weeks of differentiation. In current-clamp experiments, these cells were able to fire spontaneous action potentials after 3 weeks of induction, which were completely blocked by the sodium ion channel inhibitor TTX (400 nm, 5 min of administration). This effect was reversed by TTX washout, after which the potentials recovered in 20 min (Fig. 6e). Moreover, voltage-clamp recordings demonstrated that these converted neurons were able to show voltage-sensitive inward and outward currents, which appeared as Na^+^ and K^+^ currents according to their temporal profiles (Fig. 6f). After the addition of Na^+^ and K^+^ channel inhibitors TTX and tetraethylammonium (TEA), both Na^+^ and K^+^ currents are significantly attenuated (Fig. 6g). Importantly, we also found that approximately 52.5% of converted neurons displayed regular spontaneous discharges as typical for wild neurons. Therefore, this optimal induction system not only effectively converts hSSCs into similar morphology and phenotypes to neurons but also endues them with neuronal functionality.

### Conversation of hSSCs into DA neurons in the striatum after transplantation

**Fig. 7:**
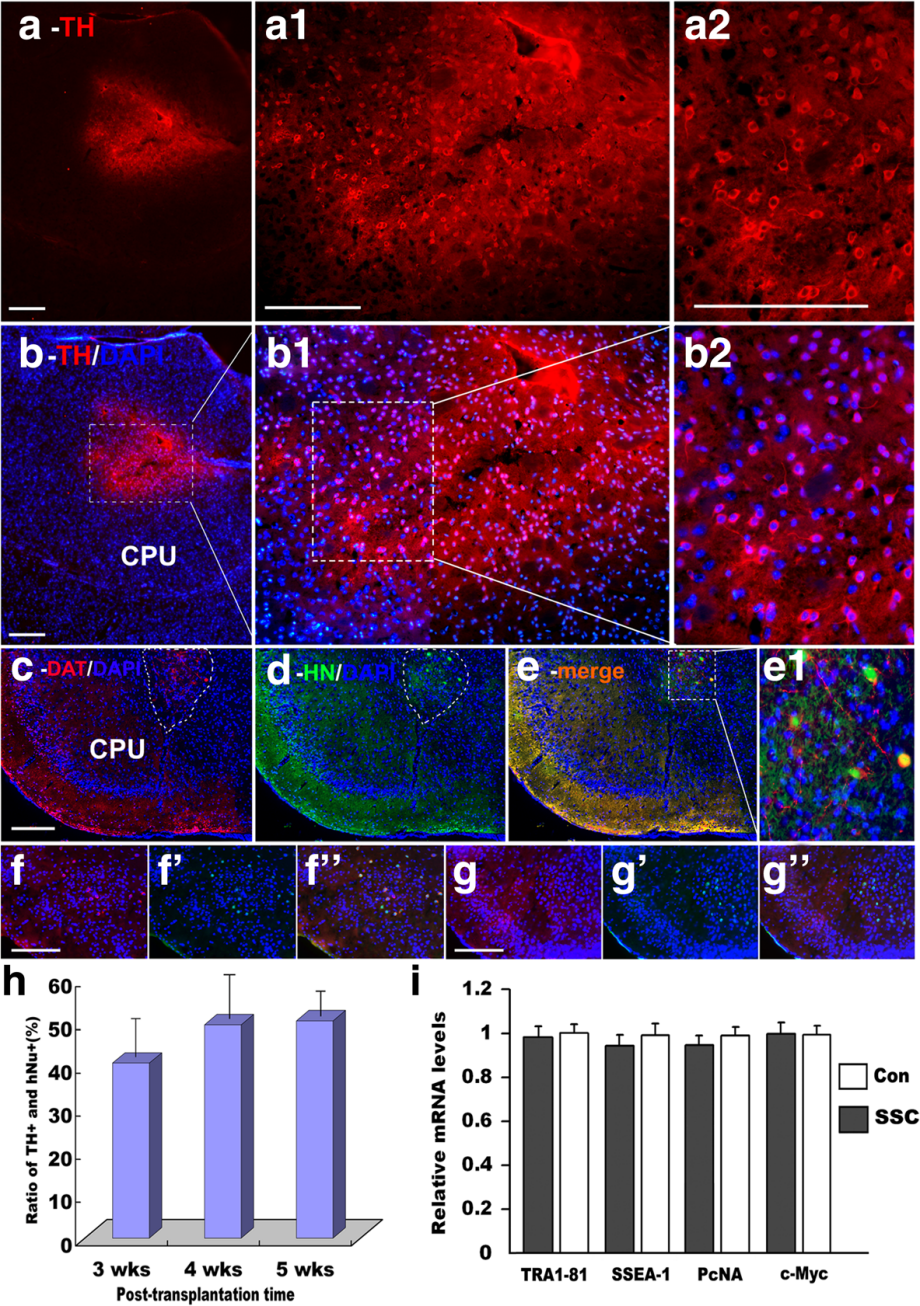
Transplantation of hSSCs into the striatum of mice and examination of tumorigenesis (**a**) TH antibody marks hSSC-derived cells integrated into the mouse brain. **a1** shows a magnification of **a**. **a2** shows a magnification of (**a1**). **b**–**b2** DAPI counterstaining of **a**–**a2**). **c**, **d** Immunofluorescence of analyses using another specific antibody targeted against DA neuron-specific DAT (**c**) and human Nuclei (hNuc) antibody (**d**). **e** Double-staining of DAT and hNuc. **e1** shows a magnification of **e**. **f**–**f”**, **g**–**g”** double-immunostaining of nestin (**f**, **g**) with cyclinD1 (**f’**, **g’**) at 1 and 2 weeks posttransplantation, respectively. **h** Quantification of the differentiation potential of DA neurons at 3, 4, and 5 weeks posttransplantation. Approximately 40–50% of the hSSC-derived cells stain TH-positive when transplanted into the striatum of mice for 3, 4 and 5 weeks. **i** Quantitative RT-PCR analysis of key genes as indicated. Scale bars = 50 μm

For in vivo monitoring of the ability of transplanted cells to survive, differentiate, integrate and migrate in appropriate sites of the striatum, we performed an immunohistological study to verify their situation over 3 weeks after transplantation. Confocal microscopy images of coronal sections stained with anti-human TH antibody showed that the transplanted cells were located in regions of engraftment site in the striatum and exhibited TH-positive reactivity (Fig. 7a, b). Excitingly, in the magnified image, many TH^+^ cells also featured extended processes with dendritic arbors (Fig. 7b2). In addition, the differentiated cells migrated from the injection sites to the outside of the striatum and projected their neurites to neighboring cells (Fig. 7a1, b1, and b2). Indeed, after week 5 posttransplantation, in vivo monitoring of the transplanted cells using double immunostaining with human DAT and hNuc antibodies demonstrated that the transplanted hSSCs in combination with induction conditions were able to survive, differentiate, and further integrate with surrounding cells (Fig. 7c, d, e, e1). To further elaborate the in vivo differentiation process, we performed double immunostaining for human nestin/cyclinD1 in the grafts. Strikingly, the minority of transplanted cells exhibited double immunostaining for human nestin/cyclinD1 at 1 week after transplantation while no double-labeled nestin and cyclinD1 cells could be seen in transplantation region after 2 weeks (Fig. 7f, g), suggesting that these transplanted hSSCs differentiated beyond 1 week without undergoing ESC process. Similar data were obtained after 3 weeks of transplantation (data not shown). In addition, the in vivo differentiation efficiency (the ratio of TH^+^ and hNu^+^) of transplanted hSSCs was evaluated within more than 15 striatal sections per animal (three animals per group) for 100 fields. Quantificative histological analysis revealed that the efficiency reached 40.5 ± 8.5%, 49.3 ± 7.9%, and 50.2 ± 10.5% at weeks 3, 4, and 5 posttransplantation, respectively (Fig. 7h). Moreover, we evaluated the safety of hSSCs by checking tumor formation at 7 weeks posttransplantation. Expression of several key genes involved in cell de-differentiation to ES, cell stemness maintenance, and tumorigenesis were not found (Fig. 7i). Together, these data indicate that the transplanted cells survived, migrated, and further converted into DA neurons without tumorigenesis.

### Assessment of sensorimotor function in mouse models of Parkinson’s disease

**Fig. 8:**
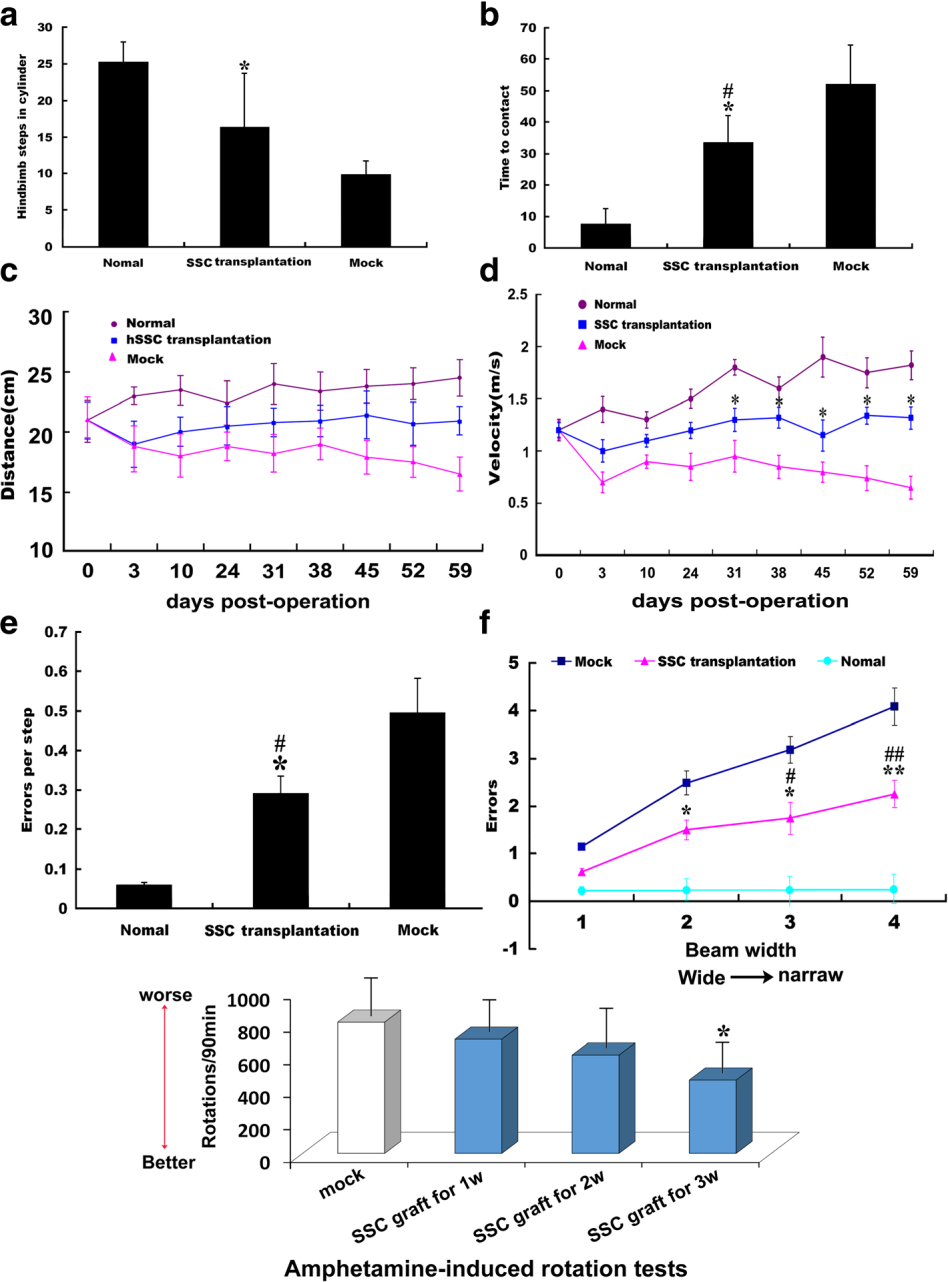
Behavioral deficit improvement after hSSC transplantation. **a** Hindlimb stepping in the cylinder in normal (*n* = 10), hSSC-transplanted (*n* = 8), and mock (saline-injected, *n* = 6) mice at 7 weeks postoperation. **b** Contact time in normal (*n* = 10) and hSSC-transplanted (*n* = 8), and mock (saline-injected, *n* = 5) mice at 7 weeks postoperation. “*” and “#” represent *p <* 0.05 compared to mock and normal, respectively. **c**, **d** Distance traveled in unit time and movement velocity in normal (*n* = 10), hSSCs-transplanted (*n* = 8), and mock (saline-injected, *n* = 6) mice at postoperation. **e** Errors per step in the challenging beam of the abovementioned mice at 7 weeks postoperation. **f** Mean errors at different beam widths. **g** The number of amphetamine-induced rotation in hSSCs groups, compared to that of the control group (mock). “*” and “**” represent *p <* 0.05 and *p <* 0.01, respectively, compared to mock. Each animal received at least five trials. “#” and “##” represent *p <* 0.05 and *p <* 0.01, respectively, compared to normal

Aside from examination of biophenotypic characteristics and physiological functions of the converted hSSCs in vivo and in vitro, behavioral measurements after hSSC transplantation are essential for the effective evaluation of potential therapeutic treatments in preclinical trials for PD. We found that all three groups of animals had almost identical sensorimotor behavioral characterization before PD model development and cell transplantation, but a remarkable behavioral change occurred in the different treatment groups (normal, hSSC transplantation, saline groups). First, a spontaneous activity test revealed that in the same line of mice treated MPTP showed a robust decrease in hindlimb stepping in the cylinder, while the reduced activity was inhibited by engrafted hSSCs. Statistical analysis indicated that there was a significant difference between the mock and hSSC transplantation groups, and no significant difference was found between the normal and hSSCs transplantation groups (Fig. 8a). Moreover, alterations in spontaneous activity to remove the label were reliably observed after hSSC transplantation. Strikingly, MPTP-treated animals engrafted with hSSCs showed significant improvement in adhesive removal performance compared with mock animals, displaying a significantly shortened time to remove the label using their forepaws. Additionally, there was a statistical difference between engrafted hSSCs of MPTP-treated and normal animals (Fig. 8b), indicating the therapeutic potential of hSSCs. Consistent with the spontaneous activity test, the gait analysis indicated that MPTP-treated mice showed a progressive decline in the walking distance during the unit time (2 min), whereas engraftment of hSSCs remarkably impeded this declining trend. Nevertheless, no significant difference was found at any of the time points posttransplantation (Fig. 8c). Likewise, the walking velocity of MPTP-treated animal was also increased by hSSC engraftment and showed an increasing tendency with prolongation of transplantation time. Strikingly, the significant increase occurred mainly beyond 4 weeks posttransplantation; however, no significant difference was observed between the hSSC transplantation and normal groups although there was an increasing trend in the walking velocity in hSSC transplantation group (Fig. 8d). In addition, analysis of challenging beam assays indicated that MPTP-treated mice showed a significant increase in errors per step compared to normal mice, while the parameter regarding errors per step was remarkably decreased by hSSC engraftment at 6 weeks posttransplantation (Fig. 8e). When animals were tested on a beam that tapered from wide to narrow, similar results were obtained (Fig. 8f), demonstrating their propensity to make errors was reduced. Additionally, the number of amphetamine-induced rotations following MPTP injection in animals transplanted pre-induced hSSCs decreased (at 1, 2, and 3 weeks posttransplantation: 899 ± 185 turns/90 min, 756 ± 215 turns/90 min, and 560 ± 235 turns/90 min, respectively) compared to control group (1033 ± 243 turns/90 min). Notably, the number of amphetamine-induced rotations significantly decreased in the animals transplanted pre-induced hSSCs for 3 weeks compared to that in control group (*p <* 0.05) (Fig. 8g).

## Discussion

The progressive degeneration and further loss of DA neurons in the midbrain is the main pathological process of PD, generally causing motor and sensory dysfunction [41, 42]. Regardless of the temporary amelioration of symptom by current therapeutic approaches (drug, gene, surgery and deep-brain stimulation, etc.), no effective treatments hitherto can cure the disease. On the basis of the properties of PD, cell replenishment therapy has been proposed as a promising treatment strategy for PD. Despite the therapeutic potential of a variety of stem cells including NSCs, their availability is extremely limited, mainly due to their individual shortcomings, including low accessibility and expansion and ethical and immune concerns [39]. Although ESCs, iPSCs, and MSCs hold great potential in regenerative medicine, their clinical application could not circumvent the risk for undesired genotoxicity, mutagenesis, and tumorigenesis besides differentiation uncertainty and ethical controversies [9–11, 43]. For this reason, finding a desirable cell-based therapy for PD is essential. Recently, advances in stem cell reprogramming studies have revealed that SSCs can transdifferentiate into neural cells, cardiovascular cells, and prostatic, uterine, and skin epithelial cells in addition to sperm lineage cells [17, 19, 22, 24, 44, 45]. This unique differentiation property is mainly attributed to both SSC-intrinsic and SSC-extrinsic factors [21]. In addition, several previous reports together with our recent studies demonstrated that SSCs have substantial advantages over other types of stem cells, such as rapid expansion, strong multipotential, and high amenability to the extrinsic environment [24, 46, 47]. More importantly, in comparison to several types of primary stem cells, SSCs have several attractive features, including no ethical concerns, lower tumorigenicity and host immune response, and wide availability [48], thus avoiding current obstacles and risks of autologous or allogenetic transplantation of SSCs. Therefore, we speculate that SSCs are likely to be the best prospective candidates for clinical applications. Nonetheless, there is not a highly successful and efficient method of reprogramming hSSCs to DA neurons.

In the present study, we developed an efficient and straightforward protocol for the conversion of hSSCs into functional DA neurons. The converted SSCs exhibited typical neuronal morphology accompanied by biochemical phenotypes of midbrain DA neurons, as well as the ability to secrete dopamine and initiate neuron-specific electrophysiology activity and calcium imaging. Following the in vitro study, the therapeutic potential of the converted cells from hSSCs was specifically supported in our in vivo experiment by behavioral improvement in the MPTP-lesioned mice after striatal cell transplantation. Safety, efficacy, and practicability will be of critical importance in developing a novel strategy for PD clinical therapy. Based on this, we tried to avoid the deficits and undesired side effects in the conventional protocol and further optimize the tedious procedure by relying on the high amenability of SSCs to the extrinsic environment. To develop a highly efficient approach for DA neuron conversion in hSSCs, apart from the delivery of traditional induction factors, such as GDNF, RA, and forskolin, OECCM and several defined components (SHH, TGFβ3, FGF8α, VPA, and SB) that effectively promote reprogramming events, including epigenetic dynamics, neurogenesis, survival, neuron subtype specification, and maturation, were supplemented. This transdifferentiation protocol for converting hSSC to DA neurons circumvents conventional drawbacks: inefficiency, tedious multistep processes, and the high risk of exogenous gene delivery. In summary, we sought to develop the hSSC induction system for future clinical consideration.

In this study, we demonstrated that this induction system is particularly amenable to giving rise to a large number of functional DA neurons as demonstrated by significant dopaminergic marker expression and functional properties after 2 and 3 weeks of in vitro induction. To gain novel insights into the molecular mechanisms underlying the neural conversion of hSSCs, we illustrate the principles of establishing this induction system, relying on the roles of each component in the conversion. Herein, OECCM was used as a critical basic component from the OEC culture supernatant. Numerous studies have demonstrated that OECs can secrete a variety of neurotrophic growth factors and cell adhesion molecules, some of which play pivotal roles in directing neural differentiation, survival, maturation, and migration and further maintenance of differentiated neuronal phenotypes [49–54]. Similar to the claim, our recent study also revealed that OECCM significantly improved adipose-derived stem cell and bone mesenchymal stem cell transdifferentiation efficiency [55, 56]; therefore, OECCM includes the induction system. The use of a defined set of factors, including SHH, GDNF, FGF8α, and TGFβ3, plays pivotal roles in neurogenesis and differentiation. In particular, some factors (e.g., SHH and FGF8α) are requisite for subtype specification of differentiating neurons during the embryonic brain development stage [57–61], since FoxA2 and Lmxla/b, two important effectors in downstream of the SHH signaling pathway, have been shown to effectively convert differentiating neural cells into DA neurons and maintain the phenotype specification [62, 63]. The presence of GDNF in the induction system mainly attributes its biofunctions to midbrain dopaminergic neurons [64, 65], such as the promotion of proliferation and specification, neurite growth, synaptic and electrophysiological maturation, soma expansion and expression of phenotype-specific proteins as well as regulating downstream effector genes. Of critical importance is our application of three components (e.g., TGFβ3, VPA and SB) required for cell lineage transdifferentiation [66, 67]. Other combined factors may also play synergistical roles in hSSC reprogramming. As a result, epigenetic switching caused by a complex interaction among these distinct molecules eventually achieves the conversion of hSSCs into DA neurons.

Although it is unclear how the minimal set of defined factors induce hSSCs to transdifferentiate into DA neurons in a short time frame, our present results have shown that our approach is efficient for generating a high number of functional DA neurons both in vitro and in vivo. To further elaborate the conversion, we assayed global transcriptome profiles and activation of several crucial proneurogenic factors before and after hSSC transdifferentiation in addition to conventional morphological traits and phenotypic characteristics. More importantly, it is imperative to determine whether the converted cells function as genuine DA neurons (e.g., synapse formation, dopamine release, neuron-specific calcium imaging and electrophysiology, and rescuing deficits after grafted in an animal model of PD). As expected, these converted hSSCs acquire cellular characteristics that assemble those of genuine DA neurons without undergoing an ESC process. Strikingly, the transdifferentiation of hSSCs toward DA neurons exhibits high efficiency due to dramatic epigenetic alterations revealed by a gain of neural cell attributes and loss of SSC fate. In particular, there is a high similarity in epigenetic genes (accounting for 88.9%) and activation of several key determinants (e.g., EN-1, Pitx3, Foxa2, Lmx1a/b, and OTX2) required for the development and specification of DA neurons [62]. The results indicate that the induction process appears to mimic the dopaminergic neurogenesis that occurs during embryonic development, albeit with limited precision. In addition, we traced the fate of engrafted hSSCs up to 4 weeks posttransplantation and found that the engrafted hSSCs could differentiate into DA neuronal lineages without forming tumors. This is likely to due to direct in vivo transdifferentiation of SSCs without ESC or intermediate precursor process, which have been strengthened by double staining for nestin and cyclinD1 in the grafts. Inspiringly, the grafted cells exhibited extensive arborization and irregular somata with robust neurite and remarkably enhanced behavioral improvement in MPTP-lesioned mice as compared to controls. Our results further revealed excellent safety and efficacy in a rodent model of PD using SSC-derived DA neurons, which may be due to direct induction without the introduction of ectopic genes. This finding has also been supported by our previous reports [20]. Thus, the present study may provide an implication for the application of SSC-based therapeutic covering a vast array of regenerative medicine disciplines.

## Conclusions

In summary, this results presented here showed that we successfully induced the differentiation of hSSCs to DA neurons using OECCM combined with a set of defined factors. The differentiated cells exhibited morphology and functionality similar to genuine DA neurons, such as spontaneous action potentials, DA release, and rescuing behavioral deficits of PD animals. Of unusual significance, the direct conversion of hSSCs to DA neurons by means of several active molecules without the instruction of exogenous genes is thus relatively safe and circumvents numerous concerns in future clinical applications. Overall, this innovative approach provides an extremely attractive solution to significant deficits in PD cell-replacement therapy. Also, this approach may serve as a general strategy for the generation of many distinct neuronal subtypes.

## Data Availability

The datasets presented in these studies are available from the corresponding author upon request.

## Abbreviations

ACSF: Artificial cerebrospinal fluid
bFGF: Basic fibroblast growth factor
BIC: Bicuculline
CD133: Prominin-1
C-myc: Proto-oncogene
CNP: 2′,3′-Cyclic nucleotide 3′phosphodiesterase
DA: Dopaminergic
DAPI: 4′-6-Diamidino-2-phenylindole
DAT: Dopamine transporter
DAZL: Deleted in azoospermia-like protein
EGF: Epidermal growth factor
EN-1: Engrailed-1
ESCs: Embryonic cells
FBS: Fetal bovine serum
FoxA2: Forkhead box protein A2
GDNF: Glial cell-derived neurotrophic factor
GFAP: Glial fibrillary acidic protein
GFRA1: GDNF family receptor alpha 1
GPR125: G protein-coupled receptor 125
HNu: Human nuclear protein
hSSCs: Human spermatogonial stem cells
iPSCs: Induced pluripotent stem cells
LIF: Leukemia inhibitory factor
Lmx1a: LIM homeobox transcription factor 1 alpha
Lmx1b: LIM homeobox transcription factor 1 alpha
MSCs: Mesenchymal stem cells
NSCs: Neural stem cells
Nurr1: Orphan member of the nuclear receptor superfamily 1
OEC: Olfactory ensheathing cell
OECCM: Olfactory ensheathing cell conditioned culture medium
OTX2: Orthodenticle homeobox 2
Pax-6: Paired box protein 6
PCNA: Proliferating cell nuclear antigen 1
PD: Parkinson’s disease
PitX3: Pituitary homeobox 3
PLZF: Promyelocytic leukemia zinc finger
SHH: Sonic hedgehog
SSEA-1: Stage-specific embryonic antigen 1
SYN: Synapsin
TEA: Tetraethylammonium chloride
TGF8α: Transforming growth factor 8 alpha
TGFβ3: Transforming growth factor beta 3
TH: Tyrosine hydroxylase
TTX: Tetrodotoxin
Tuj-1: Beta3-tubulin
UCHL1: Ubiquitin C-terminal hydrolase L1
VPA: Valproate sodium
